# Predictive factors for clinical cure in the treatment of HBeAg(−) chronic hepatitis B or compensated cirrhosis: a prospective observational study

**DOI:** 10.3389/fmed.2024.1483744

**Published:** 2025-01-09

**Authors:** Yan-Qing Lv, Ru-Hua Guo, Kun-Yu Liu, Jia-Jie Li, Hui-Fan Ji

**Affiliations:** Department of Hepatobiliary and Pancreatic Medicine, The First Hospital of Jilin University Changchun, Changchun, Jilin, China

**Keywords:** chronic hepatitis B, compensated cirrhosis, Peg-IFN-***α***-2b, clinical cure, predictive factors

## Abstract

**Background:**

Sequential or combined treatment with nucleos(t)ide analogs (NAs) and pegylated interferon alpha-2b (Peg-IFN-*α*-2b) can improve the clinical cure rate. However, its clinical application is limited due to the adverse reactions associated with IFN.

**Methods:**

A multi-center prospective observational study was conducted involving 59 NAs-treated chronic hepatitis B (CHB) patients who were treated with a combination therapy of NAs and Peg-IFN-*α*-2b for 48 weeks. Another 327 NAs-treated patients received NAs monotherapy for 48 weeks. At the end of the treatment, patients were classified into either the clinically cured group or the non-clinically cured group based on clinical efficacy. The study aimed to analyze the clinical cure rate and the predictive factors.

**Results:**

After propensity score matching (PSM), a total of 104 patients were included in the exposure and the control groups. After 48 weeks of treatment, 13 patients in the exposed group achieved clinical cure, with a cure rate of 25%. In contrast, in the control group was 1.92%. The clinical cure rate was greater in the population with CHB or compensated cirrhosis treated with sequential or combined Peg-IFN-*α*-2b and NAs than in the control group (*p* < 0.001). Patients treated with Peg-IFN-α-2b were divided into a clinical cure group and a non-clinical cure group for single-factor regression and multi-factor binary logistic regression. The results showed that baseline qHBsAg [relative ratio (RR) = 0.997, 95%CI: [0.995, 0.999], *p* = 0.031] and △TBiL (RR = 0.698, 95%CI: [0.555, 0.879], *p* = 0.002) were independent influencing factors for achieving clinical cure in patients with CHB or compensated cirrhosis.

**Conclusion:**

A lower baseline qHBsAg and decrease in TBiL at 24 weeks of treatment are independent influencing factors for achieving clinical cure. The lower the baseline qHBsAg and the higher the △TBiL levels after 24 weeks of treatment, the higher the probability of patients achieving clinical cure.

## 1 Introduction

HBV infection is a serious public health issue associated with chronic hepatitis, liver cirrhosis, and HCC (1). According to the World Health Organization (WHO), the global prevalence of HBsAg in the general population is 3.8%, with approximately 296 million chronic carriers. Approximately 820,000 people die each year due to acute or chronic liver failure, cirrhosis, or HCC caused by HBV infection (2). Currently, Entecavir (ETV), tenofovir disoproxil fumarate (TDF), tenofovir alafenamide (TAF), and pegylated interferon-alpha-2b (Peg-IFN-*α*-2b) are the preferred first-line drugs for antiviral resistance (3, 4). However, the clearance rate of HBsAg after 5 years of ETV treatment in patients with chronic hepatitis B (CHB) is less than 5%, and the rate is also low (less than 7%) in patients receiving IFN monotherapy for more than 1 year (5). The Guidelines for the Prevention and Treatment of Chronic Hepatitis B in China (version 2022) proposed that the treatment goal for certain eligible “preferential populations” is to achieve a clinical cure (6). Compared to patients with persistent surface antigen positivity, those with surface antigen clearance have a lower probability of developing cirrhosis and HCC (7, 8). In recent years, with the increasing number of CHB patients treated with IFN, there has been a growing consensus that a clinical cure can be considered a treatment goal for certain advantaged populations (9–11).

In theory, nucleos(t)ide analogs (NAs) and interferons exert different antiviral effects on HBV, and their rational combination can produce synergistic and complementary effects (12–14). Although IFN is recommended as one of the first-line treatments in all guidelines, its use has been limited due to its subcutaneous injection requirement, poor tolerance, and significant side effects. Additionally, it is contraindicated in patients with decompensated liver function, immunosuppressed states, concomitant severe disease, or pregnancy. Consequently, the use of Peg-IFN-*α*-2b in the real world remains relatively limited. Moreover, many patients treated with IFN discontinue therapy due to intolerable adverse reactions, and 40–80% of patients still do not achieve surface antigen clearance after treatment completion (5). In summary, identifying accurate predictors of a clinical cure is very important. Currently, there are few studies on the use of interferon in compensated cirrhosis. Previous research has shown that compared to CHB, there is no statistically significant difference in clinical cure rate at the end of 48 weeks of treatment (*p* > 0.05) (15).

This study included 386 patients with CHB or compensated cirrhosis, analyzed follow-up data and compared clinical cure rates between the exposed and control groups; and analyzed predictive factors for achieving a clinical cure. The aim of this study was to provide additional information for achieving a clinical cure in patients with hepatitis B.

## 2 Materials and methods

### 2.1 Study subjects

The study included a total of 386 patients with CHB or compensated cirrhosis who sought outpatient care at the Hepatology Department of either our hospital or an external hospital. The exposed group (sequential or add-on group) consisted of 59 patients who used a 1-year sequential or combined treatment with NAs and Peg-IFN-*α*-2b before September 2023. The control group (NAs group) comprised 327 CHB or compensated cirrhosis patients who sought medical attention at the Hepatology Department of our hospital from March 2023 to October 2023. The inclusion criteria were as follows: (1) diagnosis of CHB and compensated cirrhosis in accordance with the diagnostic criteria outlined in the Chinese guidelines for the prevention and treatment of CHB and cirrhosis; (2) age between 18 and 60 years; (3) CHB or compensated cirrhosis with HBV DNA (−), HBsAg ≤1,500 IU/mL, and HBeAg (−); (4) treatment with NAs for a duration of at least 1 year; (5) no IFN therapy within the past 2 years. (6) For patients receiving IFN in this study, the treatment duration should be a minimum of 1 year. The exclusion criteria were as follows: (1) history of allergy to the active ingredients, IFN-*α*, or any excipient in the drug; (2) autoimmune hepatitis or history of autoimmune disease, including immunosuppression in transplant recipients; (3) decompensated liver function; (4) uncontrolled thyroid disease; (5) severe mental illness or history of a severe mental disorder, particularly schizophrenia or depression; (6) pregnancy, potential pregnancy (female or a male partner), or lactation; (7) history of allergy to ribavirin or other NAs (such as acyclovir, ganciclovir, adenosine, etc.); (8) severe or unstable history of heart disease, including difficult-to-control cardiac conditions (myocardial infarction, heart failure, arrhythmias, etc.); (9) hemoglobinopathy (such as thalassemia, sickle cell anaemia, etc.); (10) severe renal dysfunction or a creatinine clearance rate < 50 mL/min; (11) white blood cell (WBC) count <3.0 × 10^9^/L, absolute neutrophil count <1.5 × 10^9^/L, hemoglobin (HGB) < 100 g/L, and platelet (PLT) count <90 × 10^9^/L; or (12) another condition, such as coinfection with HIV or cytomegalovirus, hepatitis C, liver cancer, severe fatty liver, etc. The study received ethical approval.

### 2.2 Study design

This was a prospective cohort study in which clinical data were collected from patient follow-ups. To address the two research objectives, the study was grouped twice. The first grouping was based on antiviral treatment methods, categorizing patients into either sequential or combination treatment with NAs and interferon, or monotherapy with NAs. The second grouping was based on whether patients achieved clinical cure at 48 weeks of treatment, categorizing patients into either clinical cure group or non-clinical cure group. Sequential treatment was defined as discontinuing the original oral antiviral medication and switching to subcutaneous injections of Peg-IFN-*α*-2b at a dose of 180 μg per week. The combination treatment consisted of oral antiviral medication and subcutaneous injections of Peg-IFN-α-2b at a dose of 180 μg/week (Peginterferon alfa-2b Injection, Xiamen Tebao Company). The study endpoint was the clinical cure of patients with CHB or compensated cirrhosis who completed 48 weeks of treatment in two groups. The patients in the control group continued oral NA treatment until the completion of 48 weeks, and their clinical cure rate was assessed. The protocol for the research project had been approved by the constituted Ethics Committee of our hospital and is in line with the Declaration of Helsinki. All persons gave their informed consent prior to their inclusion in the study.

### 2.3 Clinical and laboratory evaluations

The general information collected included age, sex, type of oral NA, and other concurrent diseases. Laboratory tests, including routine blood tests, liver function tests, quantitative HBsAg, HBV DNA, five hepatitis B markers (HBsAg, HBsAb, HBeAg, HBeAb, and HBcAb), thyroid function tests, renal function tests, blood glucose levels, lipid profiles, creatine kinase levels, and isoenzymes, were conducted at baseline and after 24 and 48 weeks of treatment. The examinations, performed at baseline, 24 weeks, and 48 weeks after treatment, included abdominal ultrasound and electrocardiography. Samples were subsequently sent for testing of routine blood markers, liver, thyroid, and renal function, blood glucose, lipids, and creatine kinase and its isoenzymes to the clinical laboratory of our hospital. Samples for testing of five markers of hepatitis B, HBV DNA, HBsAg, and hepatitis B, were sent to the Hepatology Department Laboratory.

### 2.4 Statistical analysis

Quantitative variables are expressed as the mean (±SD) or median (IQR). Group comparisons were conducted using the t-test or Wilcoxon–Mann–Whitney test. Categorical variables are represented as rates or proportions, and group comparisons were performed using the chi-square test or Fisher’s exact probability test. Propensity score matching (PSM) was applied to match patients in the exposed and control groups in a 1:1 ratio with a matching tolerance of 0.02. The chi-squared test was used to validate whether there was a difference in the overall clinical cure rate between the two groups. Simple random sampling was used to extract 100 patients from the control group of 370 patients to generate a new dataset. Univariate analysis was also conducted to examine the relationships between all the independent variables and the dependent variable. Potentially meaningless variables were filtered out (to avoid overlooking important factors, *p* values may have been appropriately relaxed). The selected independent variables were included in a binary logistic regression model to calculate the relative ratio (RR) and 95% CI for each variable. Predictive factors related to clinical cure were identified. Moreover, we constructed a receiver operating characteristic (ROC) curve and calculated the AUC to assess the predictive value of the signature. The statistical analysis was conducted using SPSS software 25, and *p* < 0.05 was considered to indicate statistical significance.

## 3 Results

### 3.1 Datasets and handling of missing values

The exposed group data showed no more than 10% missing values. Out of the 59 patients, only 5 had missing data, and the data for 54 patients were complete. In the control group, 3 variables had more than 10% missing values, 13 variables had missing values, and 8 variables were complete. Multiple imputation was applied to fill in the missing values.

### 3.2 Baseline characteristics

This study included a total of 386 patients, and the baseline characteristics are presented in Table 1. In the exposed group, 54 patients received combination therapy, and 5 received sequential therapy. The predominant antiviral medications in the exposed group were ETV, TDF, and TAF, which accounted for 64.53, 15.60, and 14.98% of the patients, respectively (with 13.56% missing data). The proportions of patients with CHB and compensated cirrhosis were 93.23 and 6.77%, respectively. The study indicated that different types of oral antiviral drugs used in sequential or combination treatment regimens had no impact on treatment outcomes (16).

**Table 1:** Baseline characteristics of the patients in the exposed and control groups before and after PSM.

Variables	Baseline			After PSM		
	NAs group (*n* = 327)	Sequential or add-on group (*n* = 59)	*P* value	NAs group (*n* = 52)	Sequential or add-on group (*n* = 52)	*P* value
Age, years, median (IQR)	49.00 (13.00)	46.00 (15.00)	0.029	46.00 (13.00)	45.50 (14.50)	0.256
Sex, male, *n* (%)	229 (70)	46 (77.97)	0.215	37 (71.15)	41 (78.85)	0.365
qHBsAg, median (IQR)	617.42 (1004.91)	227.47 (735.55)	0.001	270.37 (646.18)	311.36 (782.91)	0.520
ALT (U/L), median (IQR)	23.3 (15.20)	33.00 (31.60)	<0.001	26.60 (21.78)	31 (20.85)	0.189
AST (U/L), median (IQR)	24.60 (9.70)	28.00 (14.50)	0.095	23.35 (10.73)	26.25 (13)	0.303
TBil (umol/L), median (IQR)	15.80 (9.00)	14.00 (7.70)	0.009	13.60 (4.95)	14.30 (7.25)	0.329
ALB (g/L), median (IQR)	45.60 (3.20)	45.00 (4.60)	0.119	44.93 (2.99)	45.05 (3.05)	0.755
CHE, median (IQR)	8,143 (2091)	8,578 (2294)	0.024	8466.50 (2253.25)	8593.50 (2278.25)	0.167
WBC count (10^9^/L), median (IQR)	5.80 (1.79)	5.11 (2.02)	0.001	5.40 (1.16)	5.46 (1.72)	0.782
HBG (g/L), median (IQR)	157 (23)	155 (23)	0.121	155.50 (26.00)	155 (21)	0.603
PLT count (10^9^/L), mean (SD)	182.87 (63.30)	183.19 (54.89)	0.971	187.88 (63.59)	183.12 (54.36)	0.545
AFP, median (IQR)	3.03 (1.85)	2.79 (2.27)	0.373	2.98 (1.75)	2.83 (2.39)	0.624
Compensated cirrhosis (%)	10.09	6.77	0.426	5.77	7.69	1.000
NAs, *n* (%)						
ETV	211 (64.53)	31 (52.54)				
TDF	51 (15.60)	14 (23.73)				
TAF	49 (14.98)	6 (10.17)				
Missing	16 (4.89)	8 (13.56)				
APRI, median (IQR)						
<0.5, *n* (%)	244 (74.62)	41 (69.49)				
0.5–1.0, *n* (%)	63 (19.27)	13 (22.03)				
1.0–1.5, *n* (%)	11 (3.36)	1 (1.69)				
1.5–2.0, *n* (%)	6 (1.83)	3 (5.08)				
>2.0, *n* (%)	3 (0.92)	1 (1.69)				

In the control group, the main oral antiviral drugs used were also ETV, TDF, and TAF, which accounted for 52.54, 23.73, and 10.17% of the patients, respectively (with 4.89% missing data). There were 294 CHB patients and 33 cirrhotic patients in the control group. Statistically significant differences were observed in age; qHBsAg, alanine transaminase (ALT), total bilirubin (TBil), cholinesterase (CHE), and WBC count between the two groups, so PSM was performed at a 1:1 ratio. After PSM, 52 patients in each group were successfully matched, for a total of 104 patients. There were no statistically significant differences in variables between the two groups after PSM, as shown in Table 1.

### 3.3 Sequential or combined treatment with NAs and Peg-IFN-*α*-2b can increase the clinical cure rate in patients with CHB or compensated cirrhosis

After PSM, 52 patients were included in both the exposed and control groups. In the exposed group, 13 patients achieved a clinical cure, resulting in a clinical cure rate of 25%. In the NA monotherapy group, only one patient achieved a clinical cure, yielding a clinical cure rate of 1.92%. For the “advantaged population” among patients with CHB or compensated cirrhosis, the clinical cure rate achieved with sequential or combined treatment with NAs and Peg-IFN-*α*-2b was greater than that of NA monotherapy (*p* < 0.001). The 13 patients in the exposed group who achieved a clinical cure had an average age of 45.17 (8.25) years, with male patients accounting for 78.85%. The median of baseline qHBsAg level was 311.36 (782.91) IU/ml.

**Figure 1:**
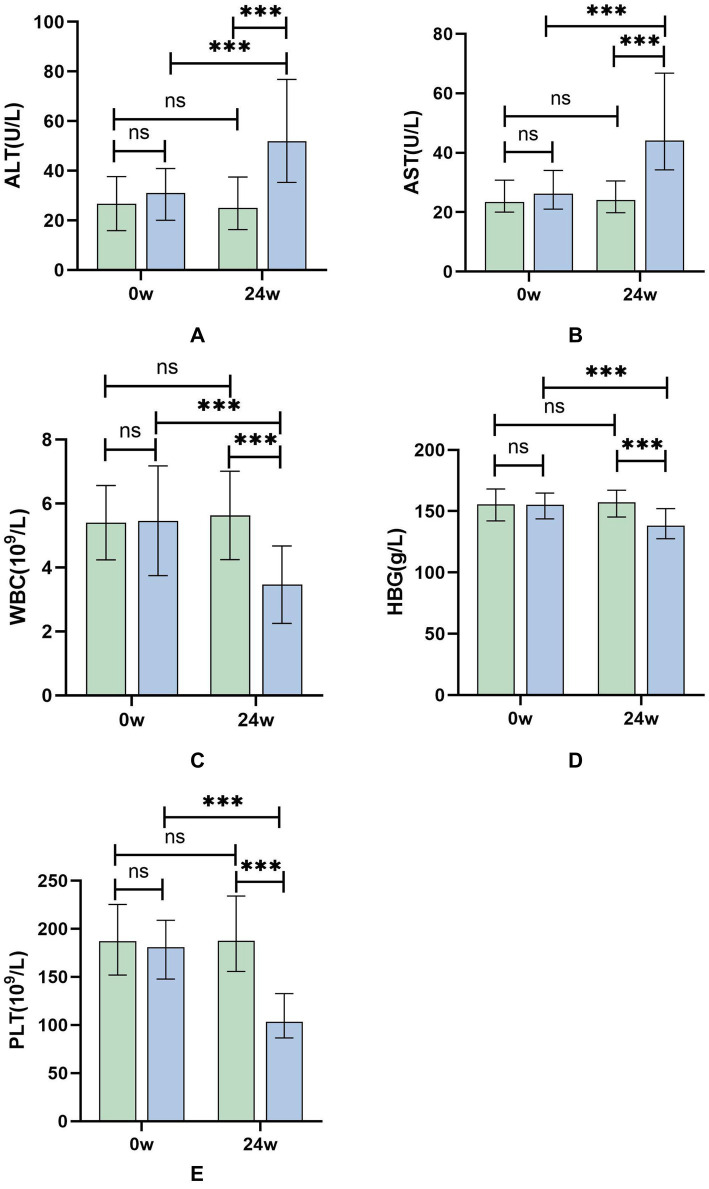
**(A–E)** Differential test results of ALT, AST, WBC, HBG, and PLT at 24 weeks of treatment for patients in the Peg-IFN-α-2b sequential or combined therapy group and the NAs group. Green: NAs group; Blue: sequential or combined therapy group. *p < 0.05, ***p < 0.001; ns, not significant.

At 24 weeks of treatment, the group receiving sequential or combined therapy with Peg-IFN-*α*-2b showed significant elevation in transaminases, along with decreases in WBC, HBG, and PLT. The changes in transaminases, WBC, HBG, and PLT in the sequential or combined therapy group compared to baseline were statistically significant (*p* < 0.001, Table 2; Figure 1), indicating that Peg-IFN-*α*-2b treatment can modulate the body’s immune function, leading to increased inflammation, thereby exerting antiviral effects. However, it also results in adverse reactions such as bone marrow suppression.

**Table 2:** Changes in transaminases and blood routine at 24 weeks of treatment.

Variables	NAs group (*n* = 52)	Sequential or add-on group (*n* = 52)	*P* value
ALT (U/L), median (IQR)	24.96 (21.18)	51.90 (41.53)	<0.001
AST (U/L), median (IQR)	24.05 (10.70)	44.10 (32.50)	<0.001
WBC count (10^9^/L), median (IQR)	5.80 (1.49)	3.01 (1.60)	<0.001
HBG (g/L), median (IQR)	157.05 (21.75)	138 (24.50)	<0.001
PLT count (10^9^/L), median (IQR)	187.62 (78.23)	103.50 (46.00)	<0.001

### 3.4 Analysis of factors associated with clinical cure

The 386 patients were divided into a case group (cured group, 16 patients) and a control group (uncured group, 370 patients). First, to ensure more reliable regression results, a random sample of 100 patients was selected from the 370 patients in the control group. Univariate analysis was subsequently conducted to examine the relationships between all the independent variables and the dependent variable. Among the 23 variables included in the univariate analysis, 7 variables showed statistical significance: qHBsAg (*p* < 0.001), ΔTBil (*p* = 0.001), ΔALB (*p* = 0.026), ΔWBC (*p* < 0.001), ΔHGB (*p* < 0.001), and ΔPLT (*p* < 0.001), sequential or add-on treatment (*p* < 0.001). ΔTBil, ΔALB, ΔWBC, ΔHGB, and ΔPLT represent the changes in variables after 24 weeks of treatment. The results of the univariate analysis are shown in Table 3.

**Table 3:** Multivariate logistic regression analysis of patients with CHB and compensated cirrhosis.

Variables	β	RR	95%CI	*P* value
Baseline qHBsAg	−0.004	0.996	0.992–0.999	0.023
△HBG	−0.102	0.903	0.818–0.997	0.042
Sequential or add-on treatment	3.802	44.775	6.150–325.966	<0.001

These 7 variables were subsequently entered into a binary logistic regression model, and the logistic regression model exhibited overall significance (*X^2^* = 60.888, *p* < 0.001). In this study, the tolerance is much greater than 0.1, and the variance inflation factor (VIF) is less than 10, indicating the absence of multicollinearity. The sensitivity of the model was 75%, and the specificity was 96%. The results showed that baseline qHBsAg (*p* = 0.023), ΔHGB (*p* = 0.042) and sequential or add-on treatment (*p* < 0.001) were found to be independent factors influencing the clinical cure rate in patients with CHB or compensated cirrhosis, while ΔTBil, ΔALB, ΔWBC, and ΔPLT showed no statistical significance. Because changes in blood routine are an adverse effect of interferon therapy, they were not considered as predictors. The results of the multivariate logistic regression are presented in Table 3.

Patients with CHB or compensated cirrhosis treated with Peg-IFN-*α*-2b were divided into a clinical cure group (14 patients) and a non-clinical cure group (45 patients). Firstly, univariate analysis was conducted to examine the relationship between all independent variables and the dependent variable. 22 variables were included in the univariate analysis, with 3 variables showing statistical significance: qHBsAg (*p* = 0.024), TBil (*p* = 0.001), and ΔTBil (*p* = 0.001). ΔTBil represents the change in variable values at 24 weeks of treatment. The results of the univariate analysis are shown in Table 4. The 3 variables were included in a binary logistic regression model. The results indicated that baseline qHBsAg (RR = 0.997, 95% CI [0.995, 0.999], *p* = 0.031) and ΔTBiL (RR = 0.698, 95% CI [0.555, 0.879], *p* = 0.002) were independent predictors for achieving clinical cure in patients with CHB or compensated cirrhosis, while TBiL did not show statistical significance (Table 5). The *β* coefficient for Baseline qHBsAg is-0.003, meaning that for each one-unit increase in qHBsAg, the likelihood of the outcome occurring is 0.997 times. The β coefficient for △TBiL is-0.359, meaning that for each one-unit increase in △TBiL, the likelihood of the outcome occurring is 0.698 times.

**Table 4:** Univariate analysis results for the clinical cure and non-clinical cure groups.

Variables	clinical cure group (*n* = 14)	non-clinical cure group (*n* = 45)	*P* value
Age, years, mean (SD)	44.36 (12.09)	45.62 (55.24)	0.630
Sex, male, *n* (%)	11 (78.57)	35 (77.78)	0.950
qHBsAg, median (IQR)	45.81 (266.96)	432.28 (787.12)	0.024
ALT (U/L), median (IQR)	33.50 (38.75)	32.50 (31.30)	0.741
AST (U/L), median (IQR)	28 (15.25)	27 (14.75)	0.558
TBil (μmol/L), median (IQR)	20.7 (9.70)	13.40 (5.75)	0.001
ALB (g/L), median (IQR)	45.95 (5)	45 (4.10)	0.812
CHE, mean (SD)	8270.21 (3375.75)	8858.44 (1749.82)	0.316
WBC count (10^9^/L), median (IQR)	5.12 (1.44)	5.11 (2.41)	0.967
HBG (g/L), median (IQR)	156.50 (18.75)	155 (23.50)	0.631
PLT count (10^9^/L), mean (SD)	203.79 (81.50)	176.78 (52.67)	0.105
Compensated cirrhosis, *n* (%)	1 (7.14)	3 (6.67)	0.321
AFP, median (IQR)	2.40 (0.93)	3.02 (2.34)	0.377
ΔqHBsAg, median (IQR)	−45.22 (267.24)	−216.49 (453.87)	0.185
ΔALT (U/L), median (IQR)	24 (49.62)	11.70 (38.65)	0.537
ΔAST (U/L), median (IQR)	10.50 (38.27)	18 (29.65)	0.273
ΔTBil (umol/L), median (IQR)	−7.15 (6.75)	−0.20 (5.30)	0.001
ΔALB (g/L), median (IQR)	−2.30 (3.31)	−1.50 (3.45)	0.460
ΔCHE, median (IQR)	41 (3071.50)	27 (1990)	0.515
ΔWBC count (10^9^/L), median (IQR)	−2.38 (1.35)	−1.93 (2.09)	0.713
ΔHBG (g/L), mean (SD)	−16.43 (12.75)	−12.33 (13.66)	0.301
ΔPLT count (10^9^/L), mean (SD)	−81.21 (69.50)	−65.52 (46.93)	0.294

**Table 5:** Multivariate logistic regression analysis of patients with CHB and compensated cirrhosis using Peg-IFN-α-2b.

Variables	*β*	RR	95%CI	*P* value
Baseline qHBsAg	−0.003	0.997	0.995–0.999	0.031
△TBiL	−0.359	0.698	0.555–0.879	0.002

### 3.5 Forest plot and ROC curve analysis

**Figure 2:**
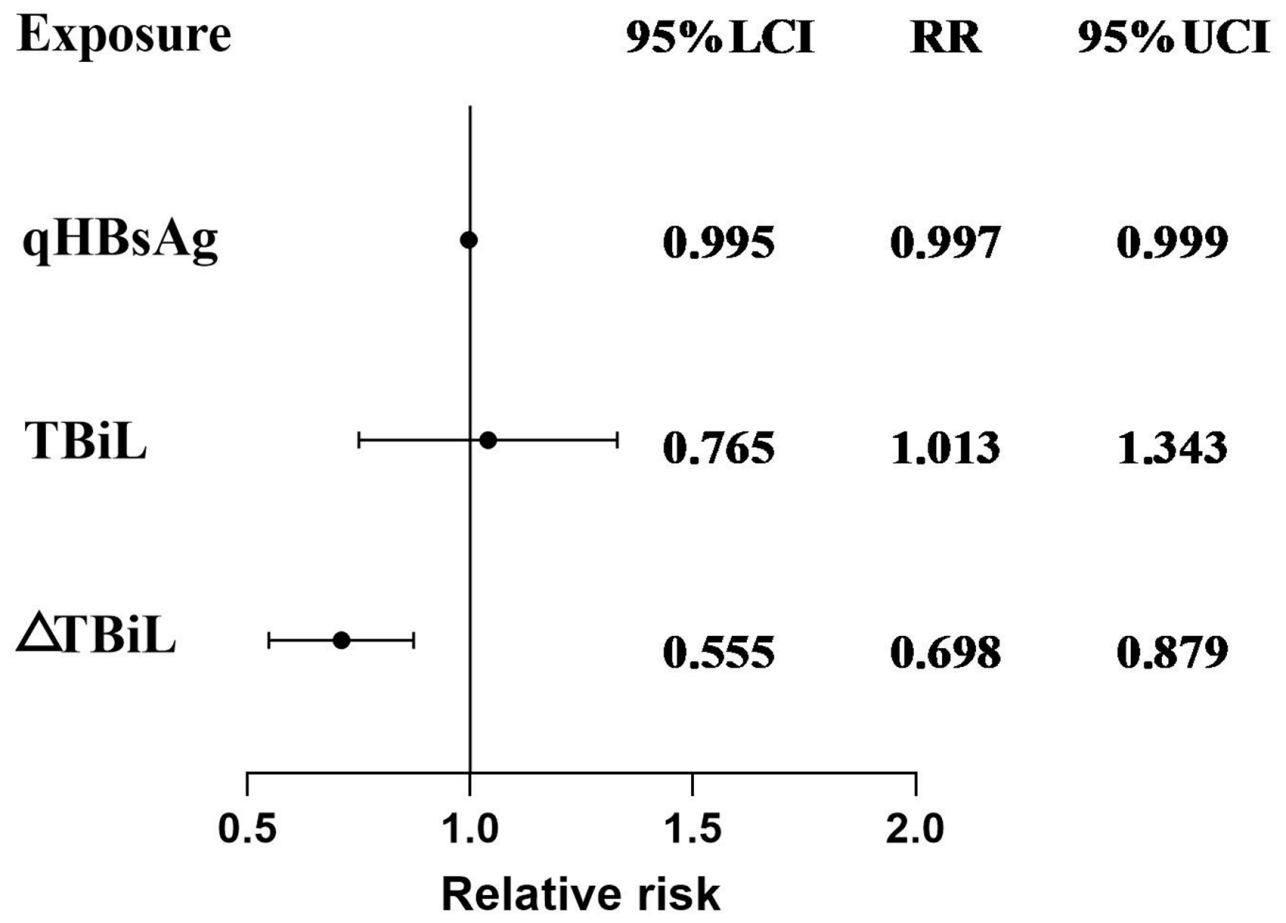
Multivariate logistic regression model forest plot results for achieving clinical cure in patients with CHB or compensated cirrhosis treated with sequential or combined Peg-IFN-α-2b therapy.

**Figure 3:**
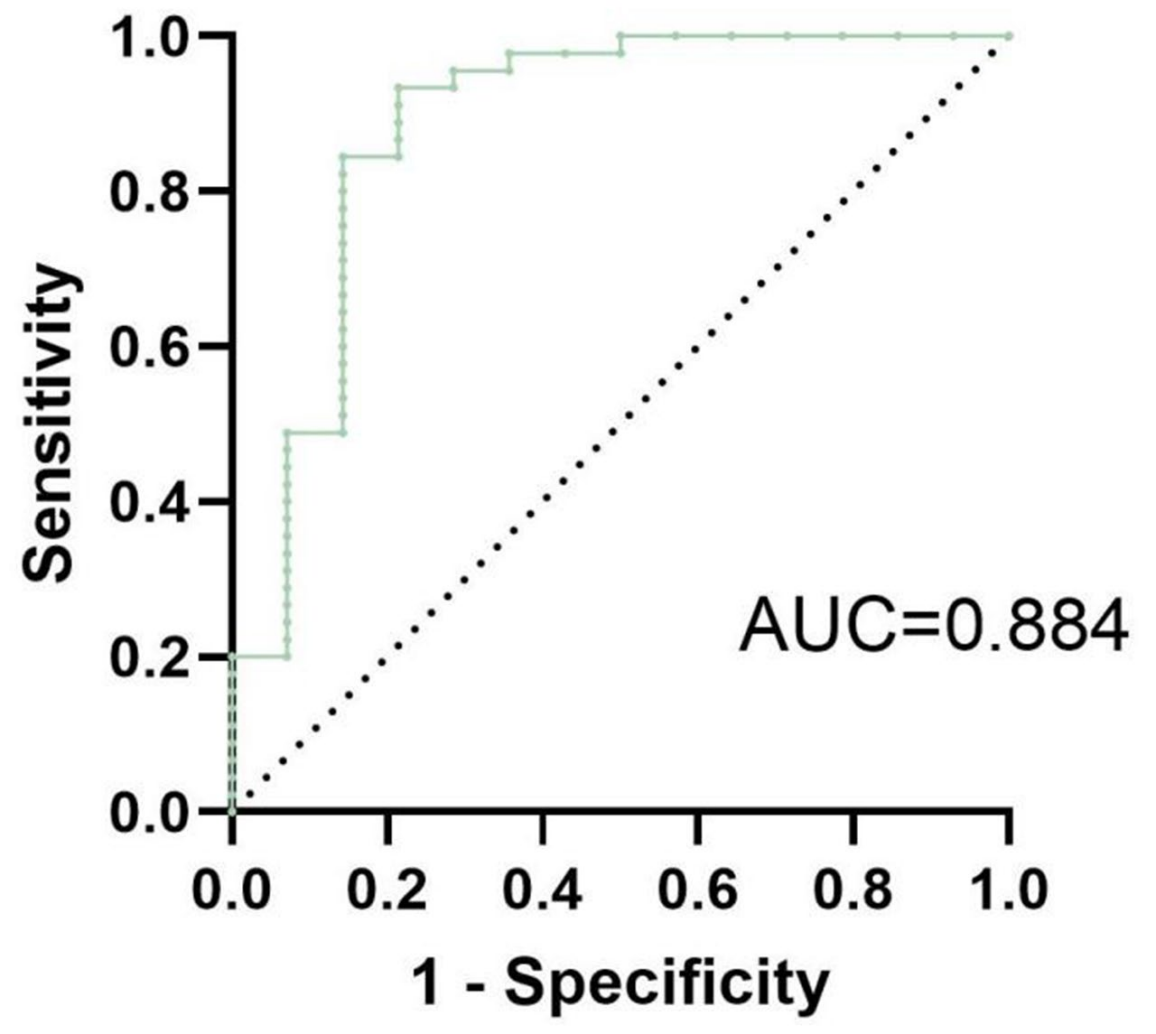
The ROC curve of the multivariate logistic regression model for achieving clinical cure in patients with CHB or compensated cirrhosis treated with sequential or combined Peg-IFN-α-2b therapy has an AUC of 0.884, with a Youden index of approximately 0.621.

To visualize the results of the logistic regression analysis, a forest plot (Figure 2) was created for the test results of the 3 variables. The results indicated that among the included variables, qHBsAg (*p* = 0.031) and ΔTBiL (*p* = 0.002) were independent factors influencing the clinical cure rate in patients with CHB or compensated cirrhosis treated with sequential or combined Peg-IFN-*α*-2b. A ROC curve was plotted (Figure 3), with an AUC of 0.884 and a Youden index of 0.621.

## 4 Discussion

NAs act on HBV DNA polymerase and reverse transcriptase, blocking the replication of HBV. DNA polymerase tends to cause errors during virus replication, and prolonged use of such drugs can lead to the accumulation of resistance, increasing potential side effects. Additionally, once the administration of these drugs is stopped, the virus is prone to rebound (7). IFNs enhance the function of immune cells, promote the expression of cytokines, induce the production of IFN-stimulated genes (ISGs) through the IFN signaling pathway, and encode various antiviral proteins that act on crucial biological processes such as HBV replication and transcription (17). This dual action of IFN plays a role in immune regulation and antiviral defence (3). IFN therapy can trigger an antiviral state in liver cells by regulating gene expression and protein translation, affecting multiple stages of the HBV lifecycle (18). Moreover, IFNs can inhibit HBV transcription and reduce the expression of viral proteins such as HBsAg by enhancing the degradation of pregenomic RNA (pgRNA) and core particles or through epigenetic modifications of covalently closed circular DNA (cccDNA) (7, 19–21).

Although sequential or combined treatment with NAs and Peg-IFN-*α*-2b can increase the clinical cure rate in patients with CHB, in practical application, many patients may not complete the 48-week course due to difficulties in tolerating the adverse reactions associated with IFN, such as extreme fatigue, hair loss, muscle pain, bone marrow suppression, depressive symptoms, and decreased gonadal function. Considering the clinical reality, when treating CHB clinically, it is first necessary to select the “advantaged population” suitable for IFN treatment. Second, accurate predictive factors need to be determined. Assessment before starting IFN therapy is recommended for patients with a lower likelihood of achieving a clinical cure and those unsuitable for IFN therapy.

The patients included in this study were recruited from two centers, and all patients were strictly screened based on the inclusion and exclusion criteria. In actual clinical treatment, patients with compensated cirrhosis also receive IFN therapy, which can reduce liver fibrosis. Therefore, patients with compensated cirrhosis were included in this study. After PSM, 52 patients were included in both the exposed and control groups, with a clinical cure rate of 25% in the exposed group. Sequential or combined Peg-IFN-*α*-2b treatment significantly improved the clinical cure rate of patients with CHB or compensated cirrhosis.

The AST-to-platelet ratio index (APRI), calculated as APRI = (AST × 100)/(ULN_AST_ × PLT), is a simple and non-invasive method for predicting liver fibrosis. Many studies have assessed the diagnostic value of the APRI. The APRI was calculated for all enrolled patients (Table 1). In the control group, the proportion of patients with an APRI >2 was 0.92%, which was lower than the actual proportion of patients with fibrosis (10.09%). In the exposed group, the proportion of patients with an APRI >2 was 1.69%, which was lower than the actual proportion of patients with fibrosis (6.77%). In this study, the diagnostic value of this indicator for liver fibrosis and cirrhosis was not high. A study by Behnaz Amernia et al. (22) suggested that the APRI is a suitable alternative for detecting significant fibrosis in NAFLD patients using FibroScan. Another study (23) indicated that the APRI has less diagnostic value than the Fibrosis-4 index (FIB-4) and NAFLD fibrosis score (NFS). However, further research is needed on non-invasive indicators and models for detecting liver fibrosis.

The 386 patients were divided into a cure group and a control group. Logistic regression analysis was performed on a randomly sampled subset of 100 patients from the control group. The results indicated that baseline qHBsAg and sequential or add-on treatment are independent influencing factors for achieving a clinical cure in patients with CHB or compensated cirrhosis. Among the 59 patients treated with Peg-IFN-*α*-2b, they were divided into a clinical cured group and a non-clinical cured group. Logistic regression analysis indicated that qHBsAg and ΔTBiL are independent influencing factors for achieving clinical cure in CHB or compensated cirrhosis patients treated with interferon. The baseline qHBsAg had a *p*-value less than 0.05, with a 95% CI for the RR value of 0.995–0.999, indicating that changes in baseline qHBsAg have a minor impact on achieving clinical cure at 48 weeks of treatment. In this study, none of the patients with compensated cirrhosis who were treated with Peg-IFN-*α*-2b developed decompensated cirrhosis. In summary, lower baseline qHBsAg and a decrease in total bilirubin levels at 24 weeks of treatment are independent factors for achieving clinical cure. Previous studies have shown that the greater the decrease in qHBsAg at 24 weeks of treatment, the higher the likelihood of achieving clinical cure. However, the data from this study do not support this conclusion. The limited number of patients treated with Peg-IFN-α-2b and those who completed the one-year treatment course may explain the difference in results and the impact on the RR value compared to other studies.

For patients who intend to undergo sequential or combined NA and Peg-IFN therapy for CHB or compensated cirrhosis, combining baseline indicators, reexamined indicators during treatment, and the presence of intolerable adverse reactions can help determine whether it is necessary to persist in completing the course of treatment when adverse reactions occur. A Chinese study showed a surface antigen clearance rate of 46.7% in patients who completed 96 weeks of treatment (24). Another prospective study by Ning Qin et al. also indicated an increased response rate in patients who completed 96 weeks of treatment (25). In this study, two patients did not achieve a clinical cure after 48 weeks but achieved surface antigen clearance after 68 weeks and 56 weeks by extending the treatment duration. These findings suggest that IFN-based treatment plans need to be individualized.

Currently, there is limited evidence from fully randomized controlled trials (RCTs) regarding clinical cure in patients with CHB. An RCT conducted by Li Jing et al. showed that IFN therapy combined with TDF treatment can improve the clinical cure rate in HBeAg-positive treatment-naive CHB patients (26). Another study by Kin Seng Liem et al. suggested that IFN therapy combined with ETV treatment in HBeAg-positive CHB patients resulted in a greater response rate (27). Another RCT indicated that lower hepatitis B core-related antigen (HBcrAg) levels and higher HBsAb levels at the treatment endpoint could be used to identify patients who might achieve a sustained functional cure after Peg-IFN-based treatment.

This was an observational cohort study of patients from two centers, including those who met the criteria for inclusion in the “advantaged population” with CHB or compensated cirrhosis. This study has several limitations. First, conducting RCTs is challenging in clinical practice, as treatment plans often follow patient preferences. Second, the inclusion of a small number of compensated cirrhosis patients in this study might have contributed to a lower clinical cure rate than that in other studies, which could have impacted the RRs in the data analysis. The conclusions of this study might also not be applicable to other regions. Additionally, as an observational study, there was a significant proportion of missing data at the 12-week follow-up, preventing an analysis of the relationship between changes in the data at 12 weeks and clinical cure. As RCTs related to clinical treatment are still limited, future research should focus on large-scale, multicenter, RCTs to provide more reliable evidence for the treatment of patients with CHB.

## Data Availability

The original contributions presented in the study are included in the article/supplementary material, further inquiries can be directed to the corresponding author.
